# Perceived Barriers to Academic Performance Among Generation Z Undergraduate Medical Students: A Cross-Sectional Study

**DOI:** 10.7759/cureus.110711

**Published:** 2026-06-12

**Authors:** Siri Chandana Putta, Anil R, Rakesh Kumar Reddy, Chitra Nagaraj, Subramaniyan P

**Affiliations:** 1 Preventive Medicine, People's Education Society Institute of Medical Sciences and Research, Kuppam, IND; 2 General Medicine, People's Education Society Institute of Medical Sciences and Research, Kuppam, IND; 3 Preventive Medicine, People’s Education Society Institute of Medical Sciences and Research, Kuppam, IND

**Keywords:** academic performance / grades, barriers, generation z, mbbs, mental health

## Abstract

Background: Generation Z is the generation of people born between 1997 and 2012. It is defined as the first generation to be born into a world with the internet, smart devices, and apps. Although digital exposure and altering learning behaviors may impact academic performance, evidence from medical students remains limited. This study seeks to identify the barriers to academic success in Generation Z medical students.

Objectives: To assess perceived barriers to academic performance among Generation Z undergraduate medical students.

Materials and methods: A cross-sectional questionnaire-based study was conducted in a private rural medical college in Andhra Pradesh, among second- and third-year undergraduate MBBS students, in August to September 2024. By the systematic random sampling method, 163 students were selected for the study. A pre-tested structured questionnaire was prepared in Google Forms (Google LLC, Mountain View, CA, USA). Participating students were met in the classroom, and the purpose of the study was explained to them. Then the Google Forms link was sent to each participant through WhatsApp (Meta Platforms, Inc., Menlo Park, CA, USA). Each participant filled out the Google Forms questionnaire and submitted the responses.

Results: A total of 163 students participated in this study with a mean age of 20.9 ± 1.43 years. About 37.4% were males and 62.6% were females. A majority, 80 (49.1%), were admitted through the government quota. The study revealed that students most frequently reported curriculum-related factors (87.7%) as perceived barriers to academic performance, followed by psychological factors (57.7%), self-related tendencies (48.5%), and external factors (36.2%). Nine students (5.5%) expressed regret at joining the MBBS program.

Conclusion: The findings suggest the need for a holistic, student-centred framework that addresses both academic and non-academic factors perceived to affect academic performance among the undergraduate medical students.

## Introduction

Generation Z is the generation of people born between 1997 and 2012. It is defined as the first generation to be born into a world with internet access, smartphone devices, and apps [1]. Generation Z students are often described as digitally engaged learners, but these characteristics should not be assumed to explain academic difficulties without direct comparative evidence. This constant exposure has reshaped the way they receive information; they demand rapid, multimodal inputs rather than traditional linear teaching methods, and it has also contributed to shorter attention spans, instant feedback expectations, and a preference for interactive, technology-integrated learning environments [2,3].

Academic performance is a broad indicator of a student’s learning outcomes and achievement, measured through examinations, assessments, and skill acquisition that reflect knowledge gain, comprehension, and application of learned material [4]. It is a key outcome influenced by cognitive, behavioral, emotional, and environmental factors, including technology use, study habits, mental health, and teaching methods. Studies show that psychosocial and contextual elements can impact academic outcomes, as sometimes they enhance engagement but often contribute to distraction and reduced performance [5].

Medical education today is a complex, evidence-based, and continuously evolving process that aims to equip future doctors with foundational biomedical knowledge and clinical skills. It also develops professional judgement, ethical reasoning, and adaptive competencies required for safe and effective patient care. It includes a structured curriculum that integrates basic sciences and clinical experiences; modern pedagogical approaches such as problem-based learning and simulation-based training to enhance competence; and rigorous assessments like objective structured clinical examinations that emphasise both knowledge and performance outcomes [6].

Evidence from studies shows that high digital engagement, especially problematic smartphone use, excessive social media activity, and multitasking, negatively affects concentration, sleep, mental well-being, and, ultimately, academic performance [7]. Most existing research has been conducted among non-medical university students, with very limited evidence focusing on medical students. Medical education has unique curriculum demands, academic pressure, and learning environments that differ from other disciplines, making it inappropriate to directly generalise findings from non-medical populations. Therefore, this study was undertaken to assess perceived barriers to academic performance among the current cohort of undergraduate medical students, who predominantly belong to Generation Z.

## Materials and methods

Study design and setting

This is a cross-sectional questionnaire-based study. The study was conducted in a private medical college located in a rural area of Andhra Pradesh. The study was conducted for a period of two months (August to September 2024).

Sampling method

A systematic random sampling method was used. A list of MBBS students was obtained from the academic office and used as the sampling frame. The sampling frame comprised all 300 MBBS students enrolled in the second (150) and third (150) years at the rural medical college during the study period. Systematic random sampling was employed for participant selection, in which the sampling interval (k) was calculated by dividing the total number of eligible students (300) by the required sample size (161). Based on this calculation, every second student from the sampling frame was selected using systematic random sampling, beginning with a randomly chosen first participant from each batch. The first participant was selected using the lottery method, and thereafter, students were chosen at regular intervals from the list until the desired sample size was achieved.

Inclusion and exclusion criteria

Inclusion Criteria

Second-year and third-year MBBS students and students who provided informed consent were included.

Exclusion Criteria

No postgraduate students included, students absent during data collection, students who were unable to complete the questionnaire adequately, students who declined participation, and those with duplicate entries in the Google Forms (Google LLC, Mountain View, CA, USA) questionnaire were excluded.

Sample size

Based on the findings of Jothula et al. [8], where 14.6% of MBBS students regretted joining MBBS, the sample size was calculated as follows.

Assumption

p is the expected prevalence = 0.146 (14.6%); q = 1 - p = 0.854; d is the margin of error or precision = 0.06 (6%); Z is the standard normal deviate at 95% confidence level, which is 1.96; n is the sample size.

Formula

$n = \frac{Z^{2} \times p \times q}{d^{2}}$

*Calculation* 

$n = \frac{(1.96)^2 \times 0.146 \times 0.854}{(0.06)^2}$ $n = \frac{3.8416 \times 0.124684}{0.0036}$ $n = 133.1 \approx 134$

After accounting for a 20% non-response rate:

$n = 134 + (20\% \text{ of } 134)$

$n = 134 + (0.20 \times 134)$

$n = 160.8 \approx 161$

Therefore, the minimum required sample size was 161. However, 163 students were selected for the study, as all eligible students who provided consent during the data collection period were included. The sample size was estimated using regret to join MBBS as a proxy indicator because no direct prior data on perceived barriers among MBBS students were available; however, this is acknowledged as a limitation because it is not identical to the primary outcome of perceived barriers to academic performance.

Study tools

A pre-tested proforma questionnaire was prepared in Google Forms to collect data. The questionnaire consisted of 44 items. The questionnaire was developed to capture perceived barriers across four predefined domains: self-related tendencies, curriculum-related factors, psychological factors, and external factors. Responses were recorded using a 5-point Likert scale ranging from strongly disagree to strongly agree. The questionnaire is provided in the Appendices.

The questionnaire development process included: (i) A small group discussion was conducted to identify common student-perceived barriers and to inform item generation for the questionnaire (based on this, four thematic areas emerged - tendency of self, psychological causes, external factors, and MBBS curriculum); (ii) the draft questionnaire was reviewed by subject experts for face and content validity; (iii) a pilot test was performed, and minor revisions were made based on participant feedback; (iv) the final questionnaire was administered for data collection using Google Forms.

The domains were operationally defined as follows: (i) Self-related tendencies (behaviors such as procrastination, motivation, and personal responsibility affecting academic performance); (ii) psychological factors(emotional and mental states, including stress and anxiety influencing academic outcomes; (iii) curriculum-related factors (academic workload, teaching methods, and syllabus-related challenges; (iv) external factors** **(environmental and social influences such as peer pressure, food habits and lifestyle).

In this study, academic success is understood as the students’ ability to meet MBBS learning requirements and progress satisfactorily through the course, while academic underperformance refers to perceived difficulty in meeting these requirements.

Barriers to academic performance are factors that reduce a student's ability to learn effectively.

For analysis, responses were coded using the 5-point Likert scale, and predefined cut-offs using mean scores were applied to classify each domain as present or absent according to the domain score.

Validation of the Proforma Questionnaire

The internal consistency of the questionnaire was assessed using Cronbach’s alpha, which was 0.78, indicating acceptable reliability. 

Method of Data Collection

Participants were approached in their respective classrooms, and the purpose of the study was explained. The Google Forms, containing the validated questionnaire along with an e-consent section, was then shared with the participants through WhatsApp (Meta Platforms, Inc., Menlo Park, CA, USA) for self-administration and data collection.

Statistical analysis of data

The data were entered in the latest version of Microsoft Excel (Microsoft Corporation, Redmond, WA, USA) and further analysed using IBM SPSS Statistics for Windows, Version 23.0 (IBM Corp., Armonk, NY, USA). Descriptive statistics were used, with categorical variables being analysed using frequencies and percentages, and continuous variables being analysed using mean and standard deviation. Bivariate analysis was performed using the Chi-square test. Likert responses were converted into domain-level categories. Each questionnaire item was scored on a 5-point Likert scale from 1 to 5. Domain scores were obtained by summing the responses to the items within each domain. The psychological domain comprised 8 items (range 8-40), the MBBS curriculum-related domain 9 items (range 9-45), the self-related tendencies domain 7 items (range 7-35), and the external influences domain 11 items (range 11-55). For bivariate analysis, the mean domain score was used as the threshold to classify participants into low and high categories, with scores below the mean considered low and scores equal to or above the mean considered high. A probability value of <0.05 was considered statistically significant. Data were grouped into categories such as curriculum-related factors, psychological factors, personal tendencies, and external factors. The results should be interpreted as associations rather than predictors or causal effects.

## Results

Sociodemographic details

Table 1 describes the sociodemographic profile of the participants. A total of 163 students participated in this study. All participants were undergraduate MBBS students from the second and third years. The majority of the students were aged above 20 years (63.8%), while 36.2% were below 20 years, with a mean age of 20.9 ± 1.43 years. Female participants constituted nearly two-thirds of the sample (62.6%), with males accounting for 37.4%. Most fathers (86.5%) and mothers (96.4%) of the participants were from non-medical professions. Regarding the type of admission secured, nearly half of the students were admitted under the Government category (49.1%), followed by the Management category (39.9%) and Non-resident Indian (NRI) category (11%).

**Table 1:** Sociodemographic details (n=163)

Variables	Categories	Frequency (%)
Age (years)	≤ 20 years	59 (36.2%)
	>20 years	104 (63.8%)
Gender	Male	61 (37.4%)
	Female	102 (62.6%)
Father's occupation	Medical profession	22 (13.5%)
	Non-medical profession	141 (86.5%)
Mother’s occupation	Medical profession	6 (3.6%)
	Non-medical profession	157 (96.4%)
Type of admission secured	Government category	80 (49.1%)
	Management category	65 (39.9%)
	Non-resident Indian (NRI) category	18 (11%)

Reasons for choosing MBBS

**Figure 1:**
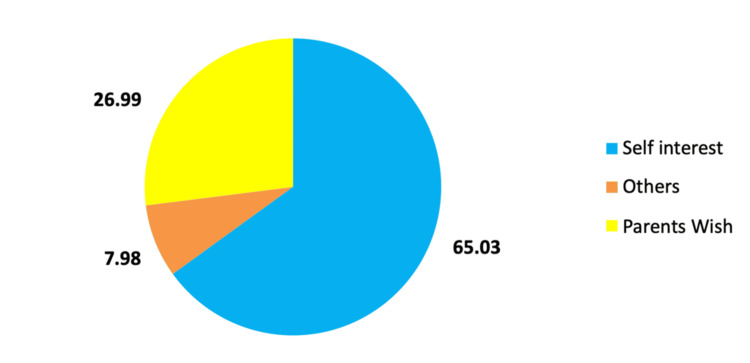
Reasons for choosing MBBS (n=163)

Figure 1 illustrates the various reasons expressed by the students for choosing MBBS as their career. Most students reported self-interest as the main reason for choosing MBBS (65.03%), while parental wish was reported as the reason by 26.99% of the participants, and the other reasons mentioned were with a smaller proportion (7.98%) for opting for the medical course.

Causes for academic underperformance

**Figure 2:**
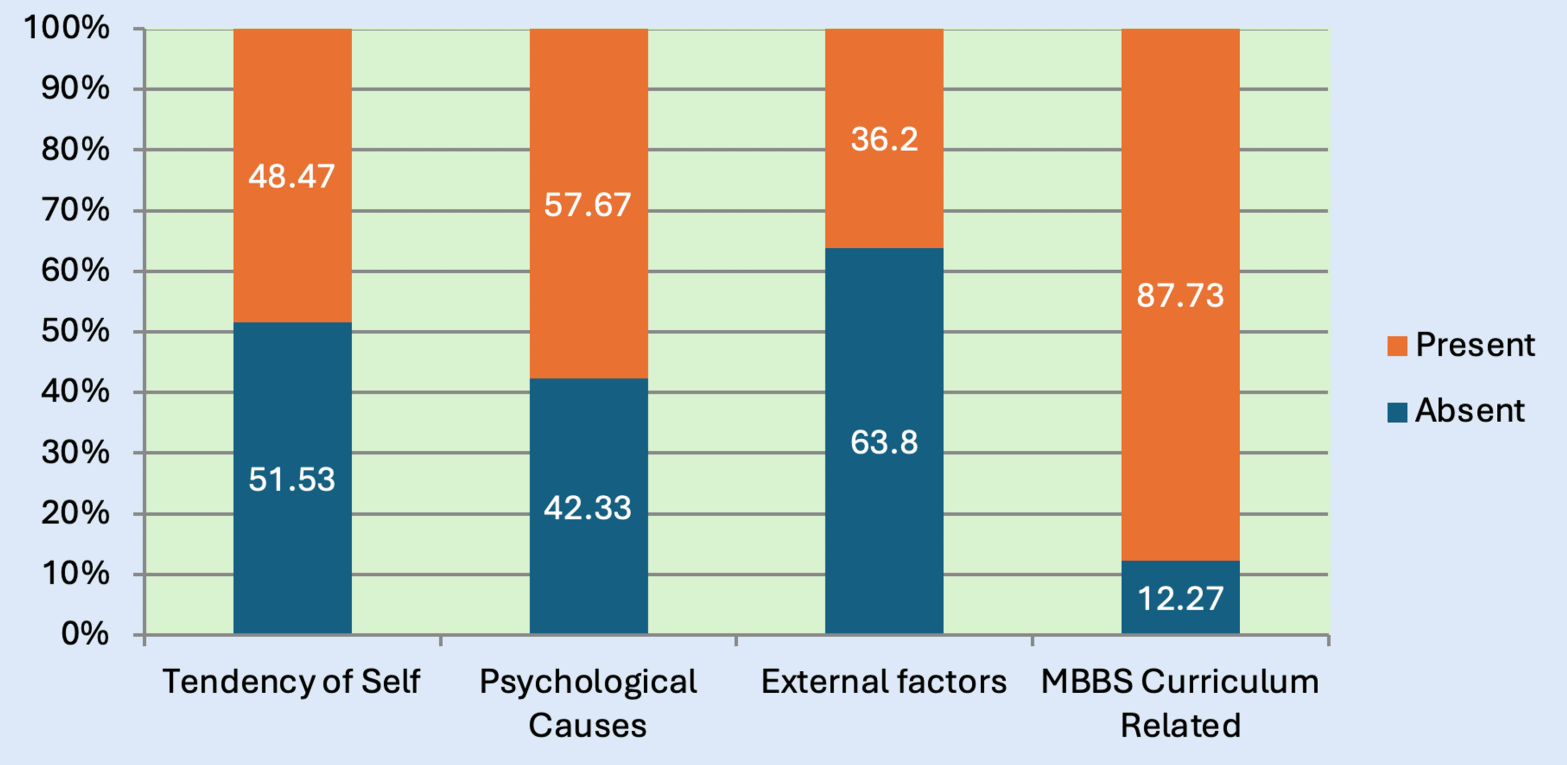
Causes for academic underperformance identified by students (n=163)

Figure 2 depicts that curriculum-related factors were most frequently reported as perceived barriers to academic performance (87.73%), followed by psychological causes (57.67%), self-related tendencies (48.5%), and external factors (36.2%).

Table 2 presents the most frequently reported causes of academic underperformance, categorized into personal tendencies, curriculum-related factors, psychological factors, and external factors. Among personal tendencies, acceptance of outcomes of personal decisions (57.66%) and procrastination (56.44%) were commonly reported. Curriculum-related factors were predominant, with insufficient holidays (84.04%) and limited understanding of foundational concepts (74.84%) being major concerns. Psychological factors included frustration due to limited sports activities (59.51%) and exam anxiety (55.83%). Among external influences, heading out for evening refreshments after 5 PM (57.67%) and food-related health issues (51.53%) were frequently reported. 

**Table 2:** Most common causes under each category (n=163)

Causes	Frequency	Percentage
Tendency of self		
Acceptance of outcomes of personal decisions	94	57.66
Procrastination on academic tasks	92	56.44
View MBBS as a step toward post-graduation	86	52.76
Distraction by social media	84	51.53
MBBS Curriculum related		
Insufficient holidays for recharging	137	84.04
Limited understanding of foundational concepts	122	74.84
Difficulty focusing due to long class hours	116	71.17
Overwhelming syllabus size	104	63.8
Psychological		
Frustration over limited sports activities	97	59.51
Exam anxiety affects study efforts	91	55.83
Hesitation to approach mentors limits guidance	71	43.56
Fear of criticism affects morale	63	38.65
External factors		
Heading out for evening refreshments	94	57.67
Food issues impact health and performance	84	51.53
Peer pressure distracts the students from academics	34	20.86
Relationship issues affect the academics	23	14.11

Association analysis

Table 3 shows the bivariate association between sociodemographic variables and tendency of self; male students demonstrated a significantly higher tendency of self compared to female students. Gender was the only variable with a statistically significant association (p-value = 0.037).

**Table 3:** Bivariate association between sociodemographic variables and tendency of self * p-value < 0.05 indicates statistically significant values.

Age (years)	Tendency of self			X^2^ value (p-value)
	Absent n (%)	Present n (%)	Total	0.2706 (0.603)
≤ 20 years	32 (54.24)	27 (45.76)	59 (100.00)	
> 20 years	52 (50.00)	52 (50.00)	104 (100.00)	
Gender				
Female	59 (57.84)	43 (42.16)	102 (100.00)	4.3441 (0.037*)
Male	25 (40.98)	36 (59.02)	61 (100.00)	
Father’s occupation				
Medical profession	9 (40.91)	13 (59.09)	22 (100.00)	1.1494 (0.284)
Non-medical profession	75 (53.19)	66 (46.81)	141 (100.00)	
Mother’s occupation				
Medical profession	3 (50.00)	3 (50.00)	6 (100.00)	0.0059 (0.939)
Non-medical profession	81 (51.59)	76 (48.41)	157 (100.00)	
Type of admission secured				
Government category	42 (52.50)	38 (47.50)	80 (100.00)	0.4077 (0.816)
Management category	34 (52.31)	31 (47.69)	65 (100.00)	
Non-resident Indian (NRI) category	8 (44.44)	10 (55.56)	18 (100.00)	
Reasons for choosing MBBS				
Self-interest	56 (52.83)	50 (47.17)	106 (100.00)	2.9303 (0.231)
Parents wish	19 (43.18)	25 (56.82)	44 (100.00)	
Others	9 (69.23)	4 (30.77)	13 (100.00)	
Total	84 (51.53)	79 (48.47)	163 (100.00)	

Table 4 shows the bivariate association between sociodemographic variables and perception of the MBBS curriculum-related issues, revealing that students whose mothers were from non-medical professions reported a significantly higher proportion of high MBBS curriculum-related issues compared to those whose mothers were from medical professions. Mother’s occupation was statistically significant (p-value = 0.001).

**Table 4:** Bivariate association between sociodemographic variables and MBBS curriculum * p-value < 0.05 indicates statistically significant values.

Age (years)	MBBS curriculum-related issues			X^2^ value (p-value)
	Absent n (%)	Present n (%)	Total	1.8809 (0.170)
≤ 20 years	10 (16.95)	49 (83.05)	59 (100.00)	
> 20 years	10 (9.62)	94 (90.38)	104 (100.00)	
Gender				
Female	11 (10.78)	91 (89.22)	102 (100.00)	0.5588 (0.455)
Male	9 (14.75)	52 (85.25)	61 (100.00)	
Father’s occupation				
Medical profession	4 (18.18)	18 (81.82)	22 (100.00)	0.8258 (0.364)
Non-medical profession	16 (11.35)	125 (88.65)	141 (100.00)	
Mother’s occupation				
Medical profession	4 (66.67)	2 (33.33)	6 (100.00)	17.1236 (0.000*)
Non-medical profession	16 (10.19)	141 (89.81)	157 (100.00)	
Type of admission secured				
Government category	10 (12.50)	70 (87.50)	80 (100.00)	0.0265 (0.987)
Management category	8 (12.31)	57 (87.69)	65 (100.00)	
Non-resident Indian (NRI) category	2 (11.11)	16 (88.89)	18 (100.00)	
Reasons for choosing MBBS				
Self-interest	14 (13.21)	92 (86.79)	106 (100.00)	0.6168 (0.735)
Parents wish	4 (9.09)	40 (90.91)	44 (100.00)	
Others	2 (15.38)	11 (84.62)	13 (100.00)	
Total	20 (12.27)	143 (87.73)	163 (100.00)	

Table 5 shows the bivariate association between sociodemographic variables and psychological causes. Age, gender, father’s occupation, mother’s occupation, type of admission secured, and reasons for choosing MBBS did not demonstrate a statistically significant relationship with psychological causes.

**Table 5:** Bivariate association between sociodemographic variables and psychological causes

Age (years)	Psychological causes			X^2^ value (p-value)
	Absent n (%)	Present n (%)	Total	0.1142 (0.735)
≤ 20 years	26 (44.07)	33 (55.93)	59 (100.00)	
> 20 years	43 (41.35)	61 (58.65)	104 (100.00)	
Gender				
Female	40 (39.22)	62 (60.78)	102 (100.00)	1.0838 (0.298)
Male	29 (47.54)	32 (52.46)	61 (100.00)	
Father’s occupation				
Medical profession	13 (59.09)	9 (40.91)	22 (100.00)	2.9263 (0.087)
Non-medical profession	56 (39.72)	85 (60.28)	141 (100.00)	
Mother’s occupation				
Medical profession	3 (50.00)	3 (50.00)	6 (100.00)	0.1501 (0.698)
Non-medical profession	66 (42.04)	91 (57.96)	157 (100.00)	
Type of admission secured				
Government category	37 (46.25)	43 (53.75)	80 (100.00)	0.9893 (0.610
Management category	25 (38.46)	40 (61.54)	65 (100.00)	
Non-resident (NRI) category	7 (38.89)	11 (61.11)	18 (100.00)	
Reasons for choosing MBBS				
Self-interest	42 (39.62)	64 (60.38)	106 (100.00)	1.2005 (0.549)
Parents wish	20 (45.45)	24 (54.55)	44 (100.00)	
Others	7 (53.85)	6 (46.15)	13 (100.00)	
Total	69 (42.33)	94 (57.67)	163 (100.00)	

Table 6 shows that the type of admission secured was significantly associated with external factors (p - value = 0.013). Students admitted under the management category exhibited a higher proportion of high external factor scores compared to those in the government and NRI categories. No other factors were statistically significant.

**Table 6:** Bivariate association between sociodemographic variables and external factors * p-value < 0.05 indicates statistically significant values.

Age (years)	External factors			X^2^ value (p-value)
	Absent n (%)	Present n (%)	Total	0.3109 (0.577)
≤ 20 years	36 (61.02)	23 (38.98)	59 (100.00)	
> 20 years	68 (65.38)	36 (34.62)	104 (100.00)	
Gender				
Female	61 (59.80)	41 (40.20)	102 (100.00)	1.8881 (0.169)
Male	43 (70.49)	18 (29.51)	61 (100.00)	
Father’s occupation				
Medical profession	15 (68.18)	7 (31.82)	22 (100.00)	0.2111 (0.646)
Non-medical profession	89 (63.12)	52 (36.88)	141 (100.00)	
Mother’s occupation				
Medical profession	4 (66.67)	2 (33.33)	6 (100.00)	0.0221 (0.882)
Non-medical profession	100 (63.69)	57 (36.31)	157 (100.00)	
Type of admission secured				
Government category	60 (75.00)	20 (25.00)	80 (100.00)	8.6181 (0.013*)
Management category	35 (53.85)	30 (46.15)	65 (100.00)	
Non-resident (NRI) category	9 (50.00)	9 (50.00)	18 (100.00)	
Reasons for choosing MBBS				
Self-interest	68 (64.15)	38 (35.85)	106 (100.00)	0.2848 (0.867)
Parents wish	27 (61.36)	17 (38.64)	44 (100.00)	
Others	9 (69.23)	4 (30.77)	13 (100.00)	
Total	104 (63.80)	59 (36.20)	163 (100.00)	

## Discussion

This study assessed perceived barriers to academic performance among undergraduate medical students, a cohort that largely belongs to Generation Z, evolving learning preferences, and increasing academic demands. The findings highlight curriculum-related factors as the most predominant contributors to success, followed by psychological factors, personal tendencies, and external influences. These findings should be interpreted cautiously because no comparison group from an earlier generation was included. Therefore, the results describe perceptions within the current undergraduate cohort rather than generation-specific differences.

In the present study, the majority of participants were aged above 20 years, with a mean age of 20.9 ± 1.43 years. This finding is consistent with several studies conducted among undergraduate medical students, reflecting the typical age at medical school enrolment [9,10]. The female predominance observed in this study (62.6%) is comparable to recent studies reporting higher female representation among medical undergraduates [9,11]. In contrast, some studies documented a higher proportion of male students [12,13].

Most participants reported that both fathers and mothers were non-medical professionals, with some similar findings from the literature showing that first-generation medical students constitute a substantial proportion of the medical student population [14]. This contrasts with a few studies that have reported a higher proportion of students from medical family backgrounds, which has been associated with early exposure to the profession and potentially different academic coping mechanisms [15]. Regarding the type of admission, nearly half of the students were admitted under the government (merit-based) category, followed by management and NRI categories. These findings align with the literature, but some studies highlighted a higher proportion of students from management categories, reflecting institutional and policy-level differences across medical colleges [16,17].

Self-interest was the main reason for choosing MBBS (65.03%) and is similar to that reported by Jothula et al. and Ibrahim Bashir et al. [8,18]. However, parental wishes accounted for 26.99% of responses, showing that family expectations continue to play a significant role in career choice, and some studies also documented stronger parental pressure than seen in our study [8,18,19].

Curriculum-related factors were the main contributors to academic success, followed by psychological factors, self-related tendencies, and external factors. Heavy academic workload, limited breaks, and difficulty understanding basic concepts were commonly reported as perceived barriers. Abdulghani et al. reported that an intensive curriculum and inadequate breaks increased stress and negatively impacted learning, whereas Satpathy P et al. also highlighted curriculum overload and insufficient rest [20,21]. In contrast, some Western studies have identified psychological stress as the primary cause of underperformance, indicating that differences in curriculum design, academic pressure, and support systems may influence students’ academic experiences [22,23]. Some curriculum-related responses may also reflect the transition to CBME rather than student generation alone.

Regarding specific domains, self-related factors such as procrastination and acceptance of outcomes of personal decisions are known to negatively affect academic performance, as studies have shown that inadequate time-management skills increase stress and reduce learning efficiency [24,25]. Psychological factors such as exam anxiety and frustration due to limited sports activities are also supported by findings from Dyrbye et al., who documented a significant correlation between anxiety, fatigue, and reduced academic performance among medical students [26]. Although external factors like food availability and related health issues were reported less often, similar findings have been noted in resource-limited settings where campus facilities influence students’ academic engagement, and in contrast, studies from well-resourced institutions report minimal impact of such factors, highlighting the importance of institutional environment [27,28].

Male students were more likely to report self-related tendencies than females in bivariate analysis [29,26]. The difference may therefore reflect internalised performance pressure, self-expectations, or independent coping behaviours among male students. Additionally, students whose mothers were from non-medical professions reported more curriculum-related difficulties, which is consistent with earlier literature that a medical family background offers better academic guidance and emotional preparedness [30].

Psychological causes, however, were not significantly associated with age, gender, parental occupation, admission type, or motivation for choosing MBBS, indicating that psychological distress may be widespread across demographic groups. Similar findings have been reported in Indian studies where stress and psychological strain were common, irrespective of demographic characteristics [10]. The type of admission showed a significant association with external factors, with management quota students experiencing high external factor scores. This may be related to financial burden, societal expectations, and performance pressure, which have been identified as important stress contributors in medical students [13].

A key strength of this study is that it focuses on Generation Z medical students, an emerging group with distinct learning behaviours, and uses a systematically selected sample with a validated, pre-tested questionnaire developed through small group discussion, expert validation, and pilot testing, which improves the relevance and clarity of the findings. However, the limitations include its cross-sectional design, which limits cause-and-effect relationships; data were self-reported, which may be influenced by recall or social desirability bias; and the study was conducted in a single rural medical college, which may limit generalisability to other settings. No comparison group from another generation was included, so generation-specific conclusions cannot be drawn. The questionnaire had acceptable internal consistency, but construct validity was not formally established. Also, the sample size calculation was not fully aligned with the primary outcome, as there was no prior data on perceived barriers among MBBS students. The possibility of sparse cells in some analyses may require interpreting bivariate associations cautiously. Future research should include multicentric and longitudinal studies to better understand causal pathways and explore the role of digital behaviour, mental health, curriculum reforms, and institutional support systems in improving academic outcomes among Generation Z medical students.

## Conclusions

This study highlights that students perceived curriculum-related, psychological, self-related, and external factors as barriers to academic performance. These findings suggest that academic performance may be affected by a combination of educational, psychological, and personal factors. Addressing these issues through curriculum reforms, strengthened academic and psychological support systems, and a more student-centred learning environment may help support student learning and academic functioning among the MBBS students.

## Appendices

Questionnaire on perceived barriers to academic performance among undergraduate medical students

Serial no: ………                                           Date of survey: …………………..

Section 1: Sociodemographic Characteristics

1. Participant ID:

2. Age:

3. Gender: Male/Female

4. Year of MBBS

5. Father’s Occupation

6. Mother’s Occupation

7. Type of admission secured: Govt./ Management Category/ NRI Category

Section 2: Reasons for Opting MBBS

  8. Reasons:

·      Self-interest

·      Fulfill parents wish

·      Parents pressure

·      Inspired from a doctor

·      To earn more money

·      Others

 9. Motivating Factors:

·      To serve the society

·      Personal interest in healthcare

·      No doctors in family

·      To make family members happy and proud

·      Respect in the society

·      To get financial stability

·      To provide health care at low cost

·      To contribute to the health research

·      Others

Section 3: Reasons for Least Attention - Perspectives

10. I think it is easy to pass MBBS examinations.

·      Strongly Disagree

·      Disagree

·      Neutral

·      Agree

·      Strongly Agree

11. I think MBBS can be learnt through online platforms.

·      Strongly Disagree

·      Disagree

·      Neutral

·      Agree

·      Strongly Agree

12. I think two months of focused reading is enough to pass the MBBS exams

·      Strongly Disagree

·      Disagree

·      Neutral

·      Agree

·      Strongly Agree

13. I think MBBS is just a step for Post-Graduation

·      Strongly Disagree

·      Disagree

·      Neutral

·      Agree

·      Strongly Agree

14. I think skills can be learnt in internship and post-graduation

·      Strongly Disagree

·      Disagree

·      Neutral

·      Agree

·      Strongly Agree

15. I am aware of the competitive world that i have to face in the future

·      Strongly Disagree

·      Disagree

·      Neutral

·      Agree

·      Strongly Agree

16. I am aware of the opportunities in future in the medical field 

·      Strongly Disagree

·      Disagree

·      Neutral

·      Agree

·      Strongly Agree

Section 4: Personal Reasons

17. I often prioritize enjoying college life (relaxing, going out, bunking classes) rather than attending lectures and studying

·      Strongly Disagree

·      Disagree

·      Neutral

·      Agree

·      Strongly Agree

18. I am frequently distracted by social media and staying up late, which impacts my study habits.

·      Strongly Disagree

·      Disagree

·      Neutral

·      Agree

·      Strongly Agree

19. I tend to procrastinate when it comes to starting my academic work

·      Strongly Disagree

·      Disagree

·      Neutral

·      Agree

·      Strongly Agree

20. I find it hard to maintain interest in my medical studies

·      Strongly Disagree

·      Disagree

·      Neutral

·      Agree

·      Strongly Agree

21. Peer pressure diverts me from academic activities

·      Strongly Disagree

·      Disagree

·      Neutral

·      Agree

·      Strongly Agree

22. Family issues divert me from academic activities  

·      Strongly Disagree

·      Disagree

·      Neutral

·      Agree

·      Strongly Agree

23. Relationship problems affect my focus on academic activities

·      Strongly Disagree

·      Disagree

·      Neutral

·      Agree

·      Strongly Agree

24. I am influenced by seniors to adopt habits that negatively affect my academic performance

·      Strongly Disagree

·      Disagree

·      Neutral

·      Agree

·      Strongly Agree

25. I struggle to stay motivated for doing my academic work

·      Strongly Disagree

·      Disagree

·      Neutral

·      Agree

·      Strongly Agree

26. Problems with the food are affecting my health and academic performance 

·      Strongly Disagree

·      Disagree

·      Neutral

·      Agree

·      Strongly Agree

27. Non-availability of food after 5 PM in the hostel mess makes me go outside for evening snacks which affects my academic performance

·      Strongly Disagree

·      Disagree

·      Neutral

·      Agree

·      Strongly Agree

Section 5: Reasons Related to MBBS Curriculum

28. I feel that my individual efforts are less

·      Strongly Disagree

·      Disagree

·      Neutral

·      Agree

·      Strongly Agree

29. I think the long class hours make it difficult for me to stay focused and study effectively

·      Strongly Disagree

·      Disagree

·      Neutral

·      Agree

·      Strongly Agree

30. I feel that the lack of holidays in the academic calendar affects my ability to recharge and study effectively

·      Strongly Disagree

·      Disagree

·      Neutral

·      Agree

·      Strongly Agree

31. I am overwhelmed by the large syllabus, making it difficult to cover all the material

·      Strongly Disagree

·      Disagree

·      Neutral

·      Agree

·      Strongly Agree

32. I think individual department's teaching methods affect my academic performance

·      Strongly Disagree

·      Disagree

·      Neutral

·      Agree

·      Strongly Agree

33. I experience learning difficulties with certain subjects

·      Strongly Disagree

·      Disagree

·      Neutral

·      Agree

·      Strongly Agree

34. I feel that poor understanding of the basics of any subject makes progressing in the subject difficult

·      Strongly Disagree

·      Disagree

·      Neutral

·      Agree

·      Strongly Agree

35. I think that relying on PowerPoint presentations (PPTs) for studying rather than books affects my deep understanding of the subject

·      Strongly Disagree

·      Disagree

·      Neutral

·      Agree

·      Strongly Agree

36. I believe that doing a presentation in the class helps me learn better

·      Strongly Disagree

·      Disagree

·      Neutral

·      Agree

·      Strongly Agree

Section 6: Psychological Reasons

37. I feel that engaging in very few social activities during my time in medical school prevents me from relaxation 

·      Strongly Disagree

·      Disagree

·      Neutral

·      Agree

·      Strongly Agree

38. I am afraid of being criticized by my peers, parents, or teachers, which affects my morale

·      Strongly Disagree

·      Disagree

·      Neutral

·      Agree

·      Strongly Agree

39. I hesitate to approach mentors or teachers which causes lack of guidance  

·      Strongly Disagree

·      Disagree

·      Neutral

·      Agree

·      Strongly Agree

40. I am prepared to face the consequences of my academic or personal decisions  

·      Strongly Disagree

·      Disagree

·      Neutral

·      Agree

·      Strongly Agree

41. I experience anxiety about the exams which affects my efforts for studying

·      Strongly Disagree

·      Disagree

·      Neutral

·      Agree

·      Strongly Agree

42. I feel that a lack of empathy towards patients affects my clinical learning

·      Strongly Disagree

·      Disagree

·      Neutral

·      Agree

·      Strongly Agree

43. I feel frustrated that the playground or sports facilities are not available every day 

·      Strongly Disagree

·      Disagree

·      Neutral

·      Agree

·      Strongly Agree

44. I regret joining the MBBS course

·      Strongly Disagree

·      Disagree

·      Neutral

·      Agree

·      Strongly Agree
